# Analyses of Stress-State-Dependent Ductile Damage and Fracture Behavior of Zirconium

**DOI:** 10.3390/ma19010081

**Published:** 2025-12-25

**Authors:** Boyu Pan, Lianghui Zhu, Zhichao Wei, Berk Tekkaya, Sophie Stebner, Sebastian Münstermann

**Affiliations:** 1 Institute of Metal Forming, RWTH Aachen University, Intzestraße 10, 52072 Aachen, Germany; boyu.pan@rwth-aachen.de (B.P.); zhichao.wei@ibf.rwth-aachen.de (Z.W.); sophie.stebner@ibf.rwth-aachen.de (S.S.); sebastian.muenstermann@ibf.rwth-aachen.de (S.M.); 2 School of Materials, University of Manchester, Manchester M13 9PL, UK; lianghui.zhu@manchester.ac.uk

**Keywords:** zirconium, stress states, fracture, stress triaxiality, lode angle

## Abstract

In this study, the fracture behavior of zirconium was investigated by a hybrid experimental and numerical simulation method. Uniaxial tensile tests were conducted on samples of various geometries, thereby covering a wide range of stress states at fracture characterized by stress triaxiality between 0.05 and 0.96 and Lode angle parameter between 0.01 and 0.95. Stress state-related parameters of each geometry were collected and used to calibrate the parameters of the modified Bai-Wierzbicki (MBW) model. With the calibrated MBW model, the fracture of zirconium can be predicted. Additionally, the fracture surfaces of the pure shear and pure tension samples were analyzed using a scanning electron microscope (SEM), revealing that shear mode dominates the failure at a low stress triaxiality range from 0 to 1/3, while ductile fracture dominates the failure at a middle stress triaxiality range from 1/3 to 1. The deep understanding of the fracture behaviors and mechanisms of zirconium under various stress states in this study contributes to the safety assessment of cladding tubes used in nuclear power plants.

## 1. Introduction

Zirconium-based alloys are widely used as cladding tube materials in nuclear power plants, especially in light-water reactors, due to their low thermal neutron absorption cross-section, high thermal conductivity, and excellent corrosion resistance [1,2,3]. During reactor operation, the internal pressure and external factors such as temperature variations, mechanical loading, irradiation, and corrosin can generate complex stress states that degrade the dimensional stability and mechanical integrity of the cladding. Under extreme conditions like loss of cooling agent (LOCA) scenarios, these effects intensify, potentially leading to failure [4,5]. Therefore, understanding the fracture behavior of zirconium under various stress states is crucial for ensuring cladding safety and reliability.

To accurately characterize and predict the fracture behavior of zirconium under various stress states, it is essential to employ a reliable and user-friendly modeling approach. Regarding this issue, numerous models have been developed, not only for zirconium but also for other metallic materials. Johnson and Cook [6,7] proposed the J-C criterion, which establishes a relationship between stress triaxiality and fracture strain, derived from tensile tests on funnel-shaped specimens with varying radii. Xu et al. [8] investigated the tensile fracture behavior of Zr-2.5Nb alloy under different stress triaxialities and calibrated the J-C model parameters based on the experimental results. The accuracy of the calibrated J-C model was subsequently validated by comparing numerical simulations with experimental results from notched specimens tested under tensile loading. Similarly, Hong et al. [9] investigated the deformation and fracture behaviors of 6061 Al alloy after reheat treatment under various stress triaxialities. Subsequently, the J-C model was used to simulate the damage of this alloy, achieving high accuracy. The J-C model can also model the strain rate dependent fracture behaviors of materials, as presented in the work of Sun et al. [10] and Öztürk et al. [11].

In another breakthrough development, Bai and Wierzbicki [12] proposed that fracture mechanisms vary depending on the mode of deformation. Their model incorporated the effects of hydrostatic pressure and Lode angle (θ) on the material damage behavior, defining a fracture locus in the space of stress triaxiality, Lode parameter, and equivalent fracture strain. Lian et al. [13] further refined this approach by coupling damage effects into the framework, resulting in the modified Bai-Wierzbicki (MBW) model. This model has gained widespread adoption due to its effectiveness in predicting ductile fracture and its straightforward implementation. Pan et al. [14] investigated the failure mechanisms of the Cr2AlC-coated Zr materials by in situ three-point bending tests, finding that the cracks initiate in the coating and subsequently propagate from the coating to the substrate. With the calibrated MBW model, the crack propagation was successfully replicated. Similar achievements in predicting material fracture behaviors can also refer to the work of Pan et al. [15]. Besides material fracture, Pan et al. [16] also applied the MBW model to predict the material’s formability and validated it with Nakajima test results. Similar success can also be seen in the work of Müller et al. [17].

Beyond macroscopic mechanical characterization and numerical modeling, a thorough understanding of fracture mechanisms at the microstructural level is essential for improving material performance, as it provides vital insights into material design, processing, and failure prevention strategies. Khandelwal et al. [18] examined the fractographic features of double-melted Zr-2.5Nb alloys and found that the presence of low-energy facets on the fracture surface, along with parallel axial splits across the specimen thickness, contributed to the alloy’s reduced fracture toughness. Xu et al. [8] performed scanning electron microscope (SEM) analyses of fractured specimens and identified that the fracture mechanism of Zr-2.5Nb alloys involves a combination of slip separation and dimple fracture. Hong et al. [19] studied the effects of hydride precipitation and reorientation on the mechanical properties of a fully recrystallized Zr-Sn-Nb cladding tube through ring tensile tests. Subsequent microstructure characterization using electron backscatter diffraction (EBSD) revealed that microvoids nucleate at the junctions between hydride platelets due to tensile deformation of the surrounding α-matrix, leading to premature fracture failure.

Based on the limitations presented in the literature review, this study aims to establish clear relationships between macroscopic stress states, fracture initiation behavior, and the underlying micro-damage mechanisms to gain a comprehensive understanding of zirconium’s fracture behavior under different loading conditions. Through a combination of experiments, numerical simulations, and scanning electron microscopy (SEM)-based fractography, the study seeks to identify the specific stress states that promote shear- or tension-dominated failure modes. Ultimately, the goal is to generate insights that support the design and assessment of zirconium components, particularly by identifying stress-state regimes that should be avoided in practical applications due to their propensity to trigger premature fracture. The study is structured as follows: Section 2 describes the investigated material and the experimental procedures. Section 3 introduces the material models used in this study, including the elasto-plasticity model and fracture criterion. Section 4 details the model parameter calibration process and presents the final material failure predictions. Section 5 presents a fractographic analysis of the fracture surfaces of the tested sample, aiming to investigate the fracture failure mechanisms. The novelty of this study lies in its hybrid experimental and numerical approach, which enables a deep investigation of zirconium’s fracture behavior under different stress states. By analyzing SEM images of fracture surfaces, the fracture mechanisms are directly correlated with the corresponding stress states, offering valuable insights into controlling zirconium fracture in practical applications.

## 2. Materials and Methods

**Figure 1:**
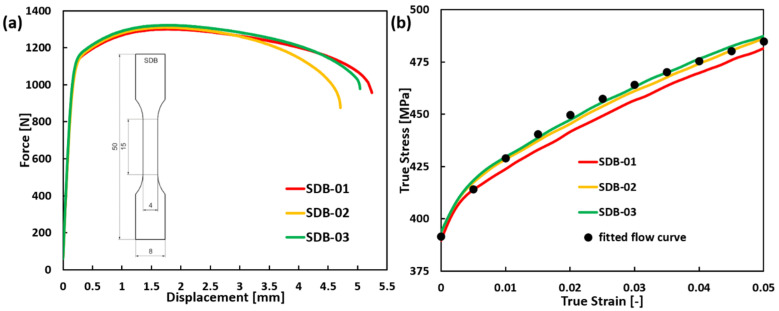
(**a**) The experimental force-displacement curves of the SDB sample and the technical drawing of the SDB sample (with a thickness of 0.7 mm); (**b**) comparison of the fitted flow curve and the experimental true stress–strain curves of SDB samples.

A zirconium sheet with a thickness of 0.7 mm and a purity of 99.5% was selected for this study. Note that zirconium has a hexagonal close-packed (hcp) crystal structure at room temperature [20]. To characterize the basic mechanical properties of the zirconium material, uniaxial tensile tests were conducted on standard dog bone (SDB) samples associated with the digital image correlation (DIC) method, qualifying the surface strain fields during the loading. A GOM Aramis system (ZEISS, Braunschweig, Germany) with a resolution of 12 Megapixels was used. These tests were performed using a universal testing machine from Zwick (Ulm, Germany) at room temperature with a global strain rate of 0.025/s. Three parallel tests were carried out to minimize the error. The design of the SDB samples and detailed experimental setup are depicted in Figure 1a, and the corresponding experimental force-displacement curve is shown in Figure 1a. The flow behavior of the investigated zirconium material was determined by fitting the parameters of the Swift hardening law to the experimentally obtained true stress–strain curves, employing a trial-and-error approach. The comparison between the experimental true stress–strain curves and the fitted flow curve is shown in Figure 1b. The final characterized mechanical properties of the zirconium material are summarized in Table 1.

**Figure 2:**
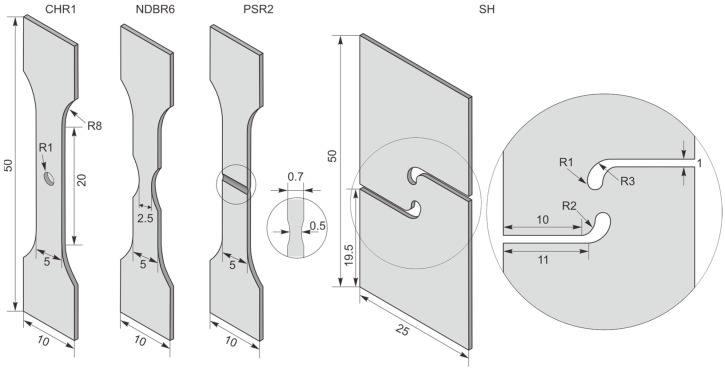
Design of the samples used to investigate fracture behaviors (all with a thickness of 0.7 mm).

To investigate the stress state effect on the fracture behavior of this zirconium material, samples with different geometries were loaded, including central hole samples with a radius of 1 mm (CHR1), notched dog bone samples with a notch radius of 6 mm (NDBR6), plane strain samples with a radius of 2 mm (PSR2), and shear (SH) samples. The selected specimen geometries span a broad range of stress states that activate different fracture mechanisms. Plane-strain and notched geometries promote void growth and coalescence under high stress triaxialities, whereas central-hole and shear geometries generate lower stress triaxiality and higher shear, conditions under which shear-driven fracture or void shearing may dominate. The chosen specimens cover this spectrum, allowing for an accurate correlation between the local stress state and the observed fracture mode. The testing conditions were identical to those when testing SDB samples. Three parallel tests were performed for each sample to minimize error. The designs of the different samples are shown in Figure 2. The final experimental results will be discussed and compared in Section 4.

## 3. Material Models

Applying appropriate models is crucial for accurately predicting material failure. This section provides a detailed overview of the elasto-plasticity model and failure model employed for zirconium, which are subsequently integrated into the numerical simulations.

### 3.1. Elasto-Plasticity Model

Tension/compression asymmetry, also referred to as the strength differential (SD) effect, is commonly observed in materials with a hexagonal close-packed (HCP) crystal structure. This effect can be attributed to: (i) activation of twinning at relatively low stress levels during in-plane compression, while it does not occur under in-plane tension [22,23]; (ii) different sensitivities to pre-existing defects, such as internal voids, pores, and surface imperfections, under tension and compression [24]. Given that the investigated zirconium material has an HCP structure, it is essential to consider the SD effect in subsequent numerical simulations. Various models have been developed to describe asymmetric plasticity and capture the SD effect, including those by Wei et al. [25], Spitzig et al. [26], Cazacu and Barlat [27], Cazacu et al. [28], Yoshida et al. [29], and Zhai et al. [30]. These models modify the von Mises yield criterion by incorporating either the first stress invariant I_1, the third stress deviator invariant J_3_, or a combination of both to account for asymmetric yielding. In this study, the Yoon2014 asymmetric model [31,32] was selected because of the availability of analytical solutions for parameter calibration. The general formulation of this model is given by:

(1) $$f=A\left[BI_{1}+{\left(J_{2}^{\frac{3}{2}}-CJ_{3}\right)}^{1/3}\right]-\sigma_{Y}\leq 0$$

(2) $$I_{1}=Tr\left(\sigma_{ij}\right)=\sigma_{1}+\sigma_{2}+\sigma_{3}$$

(3) $$J_{2}=\frac{1}{2}s_{ij}s_{ij}=\frac{1}{6}[{\left(s_{1}-s_{2}\right)}^{2}+{\left(s_{2}-s_{3}\right)}^{2}+{\left(s_{3}-s_{1}\right)}^{2}]$$

(4) $$J_{3}=\det\left(s_{ij}\right)=s_{1}s_{2}s_{3}$$

where $\sigma_{1},\;\sigma_{2},\;\sigma_{3}$ are three principal stresses of the Cauchy stress tensor σ, and $s_{1},\;s_{2},\;s_{3}$ are the corresponding principal values of the deviatoric stress tensor s. $I_{1}$ is the first invariant of the Cauchy stress tensor, while $J_{2}$ and $J_{3}$ are the second and third invariant of the deviatoric part of the Cauchy stress tensor. The yield strength of the material is denoted by $\sigma_{Y}$. The material constants B and C control the influence of pressure and the third invariant on material yielding, respectively. The constant A is related to the strain hardening properties of the material and is computed as:

(5) $$A=\frac{1}{B+{\left(\frac{1}{3\sqrt{3}-\frac{1}{27}C}\right)}^{1/3}}$$

For pressure insensitive materials, the yield function simplifies to the isotropic Cazacu and Barlat [27] model by setting B = 0. In this case, the material constants *A* and *C* can be determined as follows:

(6) $$A=\frac{\sqrt{3}\varphi_{C,0}}{{\left(1+\frac{{\varphi_{C,0}}^{3}-1}{{\varphi_{C,0}}^{3}+1}\right)}^{1/3}}$$

(7) $$C=\frac{3\sqrt{3}}{2}\cdot \frac{{\varphi_{C,0}}^{3}-1}{{\varphi_{C,0}}^{3}+1}$$

where $\varphi_{C,0}=\frac{\sigma_{T,Y}}{\sigma_{C,Y}}$. $\sigma_{T,Y}$ $\sigma_{C,Y}$ are uniaxial tensile yield stress and uniaxial compressive yield stress, respectively.

Equation (1) can also be extended into an anisotropic form via a linear transformation, as detailed in the studies by Yoon [32] and Hu and Yoon [31]. The applicability of the analytical Yoon2014 model has been validated in the work of [33,34].

### 3.2. Fracture Criterion

Numerous ductile fracture models have been proposed to describe the influence of stress state on ductile damage and fracture, as presented in the works of Besson [35], Pineau et al. [36], Benzerga et al. [37], and Tekkaya et al. [38]. Among them, the modified Bai-Wierzbicki (MBW) model, introduced by Lian and Sharaf [13], has been widely adopted for its effectiveness in predicting ductile failure across various materials [14,39,40]. In this model, damage induced softening after the damage initiation is quantified by a scalar parameter D:

(8) $$f=\left(A,B,C|I_{1},\;J_{2},J_{3}\right)-\left(1-D\right)\sigma_{Y}\leq 0$$

As shown above, the Yoon2014 yield model is integrated into the MBW model. $\sigma_{Y}$ is the flow stress formulated by the selected hardening laws, according to the features of the flow curves [41]. The Swift hardening law was selected in this study. In the MBW model, the stress states are characterized by two independent parameters: stress triaxiality η and the Lode angle parameter $\bar{\theta }$, which are expressed in terms of stress invariants. For proportional loading, the damage initiation locus (DIL) is described by a critical plastic strain ${\bar{\varepsilon }}_{ddi}$ as a function of the stress states. When the DIL is reached during plastic deformation, damage initiation begins.

(9) $${\bar{\varepsilon }}_{ddi}\left(\eta ,\bar{\theta }\right)=\left(D_{1}\cdot e^{-D_{2}\cdot \eta }-D_{3}\cdot e^{{-D}_{4}\cdot \eta }\right)\cdot {\bar{\theta }\;}^{2}+D_{3}\cdot e^{-D_{4}\cdot \eta }$$

(10) $$\eta =\frac{I_{1}}{3\sqrt{3\cdot J_{2}}}$$

(11) $$\theta =\frac{1}{3}\cdot {cos}^{-1}\cdot \left(\sqrt{27/4}\cdot J_{3}\cdot {J_{2}}^{-3/2}\right),\;0\leq \theta \leq \frac{\pi }{3}$$

(12) $$\bar{\theta }=1-6\cdot \frac{\theta }{\pi },-1\leq \bar{\theta }\leq 1$$

where $D_{1}\sim D_{4}\;$ are material parameters calibrated from experiments.

Since ideal stress states are rarely maintained in real cases, the average stress triaxiality $\eta_{avg}$ and the average Lode angle parameter ${\bar{\theta }}_{avg}$ are used to account for the change in stress states during plastic deformation. Additionally, a damage initiation indicator $I_{dd}$ is employed for non-proportional loading conditions, accumulating throughout plastic strain evolution [42]. Damage initiation occurs when $I_{dd}$ reaches unity. A cutoff value of −1/3 for η is assumed for the investigated material [43], below which neither damage initiation nor evolution occurs.

(13) $$\eta_{avg}=\frac{1}{{\bar{\varepsilon }}^{p}}\int_{0}^{{\bar{\varepsilon }}^{p}}\eta \;d{\bar{\varepsilon }}^{p}$$

(14) $${\bar{\theta }}_{avg}=\frac{1}{{\bar{\varepsilon }}^{p}}\int_{0}^{{\bar{\varepsilon }}^{p}}\bar{\theta }\;d{\bar{\varepsilon }}^{p}$$

(15) $$I_{dd}=\int_{0}^{{\bar{\varepsilon }}^{p}}\frac{1}{{\bar{\varepsilon }}_{ddi}\left(\eta ,\bar{\theta }\right)}\;d{\bar{\varepsilon }}^{p}$$

Based on the energy dissipation theory, a linear damage evolution law is employed for simplicity. The damage evolution is governed by the equivalent stress at the damage initiation point ${\bar{\sigma }}_{ddi}$ and the parameter $G_{f}$. Similarly to the DIL, a ductile fracture locus (DFL) is defined with four additional parameters $D_{5}\sim D_{8}$, with the ductile fracture indicator $I_{df}$ describing the damage accumulation. A detailed formulation of the MBW model is provided by [44,45,46,47].

(16) $${\bar{\varepsilon }}_{df}\left(\eta ,\bar{\theta }\right)=\left(D_{5}\cdot e^{{-D}_{6}\cdot \eta }-D_{7}\cdot e^{-D_{8}\cdot \eta }\right)\cdot {\bar{\theta }\;}^{2}+D_{7}\cdot e^{-D_{8}\cdot \eta }$$

(17) $$I_{df}=\int_{{\bar{\varepsilon }}_{di}^{p,c}}^{{\bar{\varepsilon }}^{p}}\frac{d{\bar{\varepsilon }}^{p}}{{\bar{\varepsilon }}_{df}\left(\eta ,\bar{\theta }\right)-{\bar{\varepsilon }}_{ddi}\left(\eta ,\bar{\theta }\right)}\;d{\bar{\varepsilon }}^{p}$$

(18) $$D=\left\{\begin{array}{ccc} 0\;\;\;\;\;\;\;\;\;\;\; & I_{dd}<1\;\;\;\;\;\;\; & \\ \frac{{\bar{\sigma }}_{di}^{c}}{G_{f}}\left({\bar{\varepsilon }}_{df}^{p}-{\bar{\varepsilon }}_{di}^{p}\right)\cdot I_{df}\;\;\;\;\;\;\;\;\;\;\;\; & I_{dd}\geq 1\;\;\;\;\wedge & I_{df}<1\\ 1\;\;\;\;\;\;\;\;\;\;\; & I_{dd}\geq 1\;\;\;\;\wedge & I_{df}\geq 1 \end{array}\right.$$

Note that, in this study, the MBW model is employed as an uncoupled damage model by only using the DIL. Thus, fracture is described by $I_{dd}=1.$

## 4. Results and Discussion

Following the calibration of the fundamental mechanical properties of the investigated zirconium, numerical simulations were conducted to facilitate the calibration of model parameters. Once the model was calibrated, it was subsequently used to predict material failure.

### 4.1. Calibration of the Model Parameters

**Figure 3:**
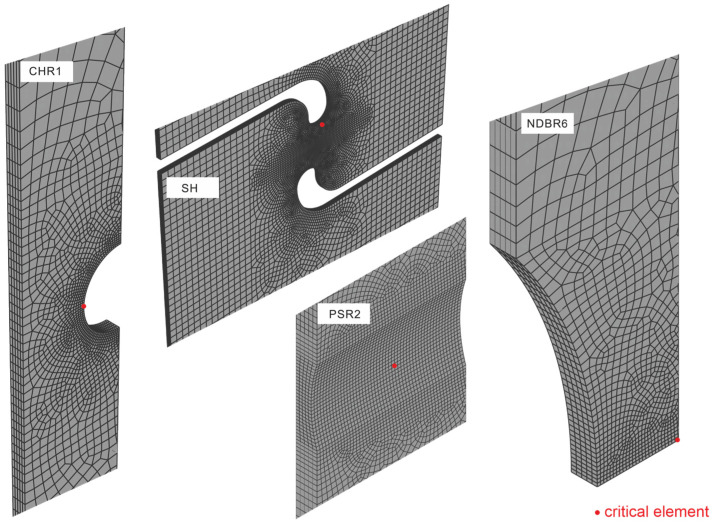
Finite element models of tested sample geometries constructed in ABAQUS representing the meshing strategy and the critical element (in red) with the highest plastic strain value. The finest mesh size is 0.05 mm. The total number of elements are 13,965, 85,100, 33,485 and 17,325 for CHR1, SH, PSR2 and NDBR6, respectively. Note that the mesh is shown only for the region of interest to better display the mesh size; the full geometry mesh for each of the samples shown in Figure 2 is used in the numerical simulation.

Finite element (FE) simulations were conducted to extract the stress state related parameters of the critical zirconium element (the element with highest plastic strain value) at the moment of fracture. Given the symmetry of the samples, only half of the model was constructed in ABAQUS. The mesh of the geometrical models is shown in Figure 3. The critical element experiencing the highest plastic strain value for each geometry is highlighted in red. The zirconium samples were meshed using eight-node three-dimensional continuum elements with reduced integration (C3D8R). As boundary conditions, the samples were fixed at the bottom and z-symmetry boundary conditions were applied on the symmetry plane. The displacements were prescribed at the top of the samples.

As discussed in Section 3.1, the analytical Yoon2014 model required the calibration of three parameters, denoted as A, B and C. Since zirconium is considered pressure insensitive, the parameter B is set to zero. However, due to the challenges associated with performing uniaxial compression tests on thin sheet materials, parameters A and C were calibrated using the uniaxial tensile and compressive yield stresses of a similar material Zircaloy-4, reported in the literature [21]. According to [21], the uniaxial tensile and compressive yield strengths of Zircaloy-4 are approximately 430 MPa and 450 MPa, respectively. Based on these values, the calibrated elasto-plasticity model incorporating the analytical Yoon2014 criterion for the investigated zirconium is expressed as follows, with parameters A and C equal to 1.7 and −0.17, respectively. It should be noted that the assumption of pressure independent yielding may lead to uncertainties in the simulation results. In particular, small deviations between the tension–compression asymmetry of investigated zirconium sheet and the reported values for Zircaloy-4 could slightly affect the predicted stress response under high hydrostatic compression. However, since the stress states investigated in this study are predominantly tensile or shear-dominated (see Table 2) and do not approach regimes where pressure sensitivity becomes the governing factor, the resulting influence on the calibration of the fracture model is expected to be limited.

(19) $$f=1.7\cdot \left[{\left(J_{2}^{\frac{3}{2}}-(-0.17\cdot J_{3})\right)}^{1/3}\right]-\sigma_{Y}$$

This calibrated elasto-plasticity model was subsequently integrated into the simulations.

During the simulation, a dynamic explicit step was employed with a total time period of 0.01 s and a time scaling factor of 1. The linear bulk viscosity parameter was set to 0.06, and the quadratic bulk viscosity parameter was assigned a value of 1.2. To calibrate the fracture model material parameters, the failure criterion was not activated in this stage of the numerical simulations.

**Figure 4:**
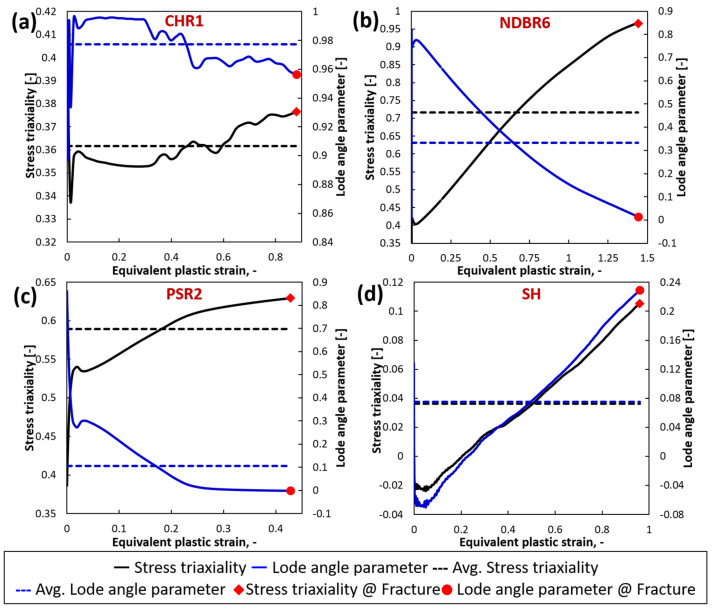
The evolution of stress triaxiality and the Lode angle parameter from the critical element with the increasing strain for (**a**) CHR1, (**b**) NDBR6, (**c**) PSR2, and (**d**) SH specimens.

Upon completion of the simulations, the local stress and strain variables of the critical elements in each model, including equivalent plastic strain (PEEQ) and average stress triaxiality $\eta_{avg}$ and ${\bar{\theta }}_{avg}$, were extracted at the displacement corresponding to the onset of fracture. These results are summarized in Table 2. Figure 4 illustrates the evolution of the stress triaxiality η and the Lode angle parameter $\bar{\theta }$ from the critical element with the increasing plastic strain. For each specimen, the actual stress triaxiality η and the actual Lode angle parameter $\bar{\theta }$ at fracture initiation are also marked in the graph. Across all tested samples, $\eta_{avg}$ falls within the range of −0.02 to 1, while ${\bar{\theta }}_{avg}$ range from −0.08 to 1, covering a relatively wide range of stress states. For the CHR1 sample, both η and $\bar{\theta }$ experience pronounced changes as plastic strain increases until fracture occurs. In the case of NDBR6 and PSR2, η generally increases with increasing plastic strain, whereas $\bar{\theta }$ follows a decreasing trend. Conversely, for the SH sample, both η and $\bar{\theta }$ increase with increasing plastic strain. These phenomena highlight the necessity of considering $\eta_{avg}$ and ${\bar{\theta }}_{avg}$ to account for the change in stress state during deformation.

**Figure 5:**
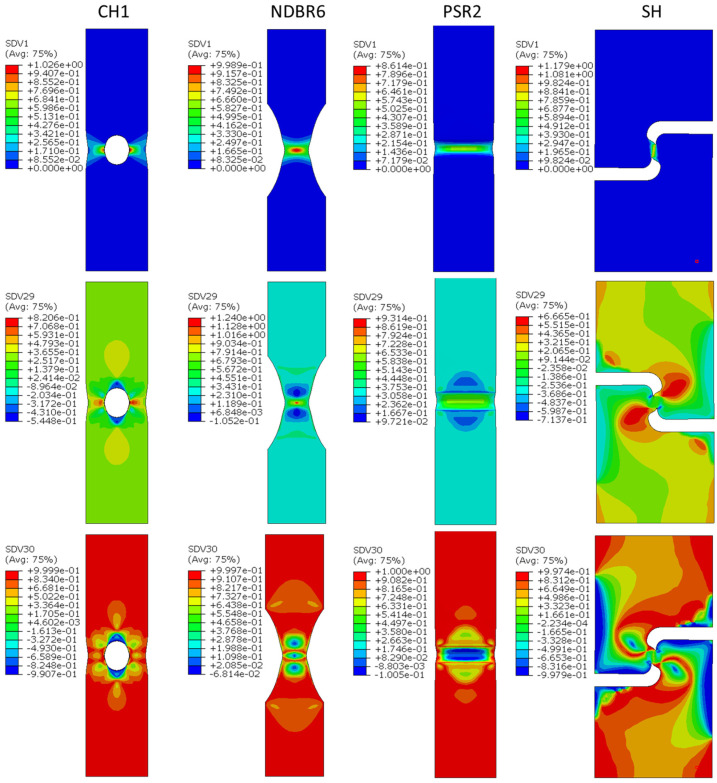
The distribution of equivalent plastic stain (SDV1), stress triaxiality (SDV29) and Lode angle parameter (SDV30) prior fracture in the samples CH1, NDBR6, PSR2 and SH viewed on the free surfaces of the samples.

Figure 5 shows the stress state in terms of stress triaxiality (SDV29) and the Lode angle parameter (SDV30) for the tested samples, as well as the equivalent plastic strain (SDV1) prior to fracture. The numerical results clearly demonstrate how the chosen specimen geometries generate distinct stress states. In the CH1 central-hole specimen, plastic strain (SDV1) develops in a broad, symmetric ring around the hole, accompanied by moderate stress triaxiality (SDV29) and Lode angle parameter values characteristic of a biaxial tensile condition. In the NDBR6 notched dog-bone geometry, plastic deformation becomes more localized along the ligament, and the notch produces elevated stress triaxiality together with a transition toward mixed tension–shear Lode parameter values. The PSR2 plane-strain specimen shows the strongest confinement of plastic strain, with the highest stress triaxiality concentrated immediately ahead of the notch root and Lode angle parameter values approaching those of axisymmetric tension, consistent with a high-constraint void-growth dominated stress state. In contrast, the shear (SH) specimen exhibits a markedly different behavior: plastic strain localizes along diagonal shear bands rather than in the ligament center, while stress triaxiality remains low throughout the specimen. The Lode angle parameter indicates a shear-dominated stress state with values near the limits associated with shear failure. Together, these four geometries span a wide and representative range of stress triaxialities and Lode angle parameters, from high-constraint plane-strain tension to low stress triaxiality shear, providing a comprehensive basis for assessing the stress-state dependence of fracture in the zirconium material.

**Figure 6:**
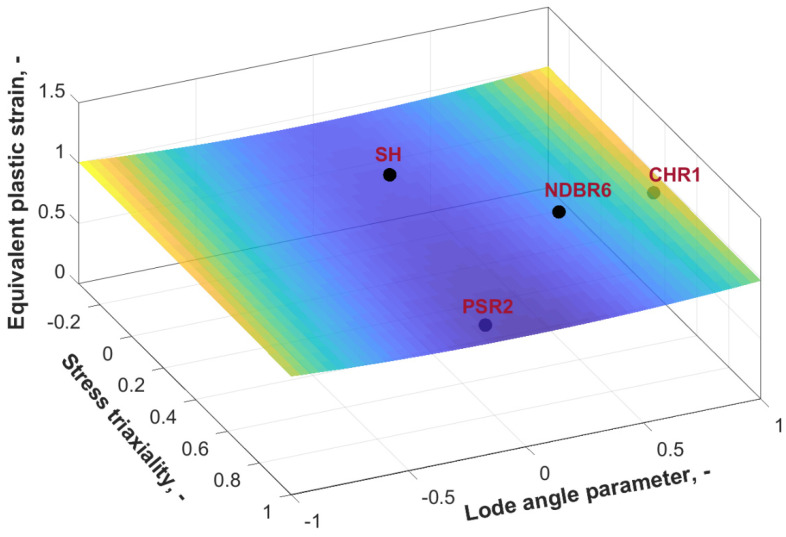
Calibrated damage initiation locus for zirconium corresponding to the onset of fracture. (For interpretation of the references to color in this figure legend, the reader is referred to the web version of this article).

With a primary focus on accurately capturing the fracture initiation behavior of zirconium samples, significant emphasis was placed on identifying the damage initiation parameters for them. In this regard, the uncoupled damage mechanics approach was adopted, wherein a small value of $G_{f}$ (100 MPa) was selected, and the difference between DIL and DFL was ignored. Based on the values summarized in Table 2, the damage parameters in the strain-based DIL were optimized for zirconium, as detailed in Table 3. To calibrate the damage model parameters $D_{1}\sim D_{4}$ for zirconium, the collected $\eta_{avg}$, ${\bar{\theta }}_{avg}$, and the corresponding PEEQ values from Table 2 were utilized as input data. The calibration process was conducted using the curve fitting tool in MATLAB R2021b, where Equation (9) for the DIL was implemented as a custom equation, with η, $\bar{\theta }$, and PEEQ assigned as the x-, y-, and z-variables, respectively. The trust-region algorithm was employed for the parameter optimization, ensuring robust convergence. Furthermore, the lower bounds for $D_{1}\sim D_{4}$ were constrained to zero to maintain a physically meaningful and well-defined DIL shape, as suggested in previous studies [13,14]. To mitigate numerical sensitivity, the finite-differencing step limits were constrained, with DiffMaxChange and DiffMinChange defined as 0.1 and 1 × 10^−8^, respectively. Both the function convergence (TolFun) and step size tolerance (TolX) were both restricted to 1 × 10^−6^. The final constructed DIL is illustrated in Figure 6, along with experimental results for four different geometries for comparison. The x-, y-, and z-axes correspond to η, $\bar{\theta }$ and PEEQ, respectively. The three-dimensional surface represents the critical strain required for damage initiation under the ideal proportional loading conditions in the general stress state space. In cases of proportional loading, the experimental fracture points should align with the damage initiation locus. However, due to the non-proportional loading paths experienced by the material during tensile tests, variations in the degree of alignment between the experimental data points and the fitted surface were observed.

### 4.2. Prediction of Fracture Behavior of Zirconium

**Figure 7:**
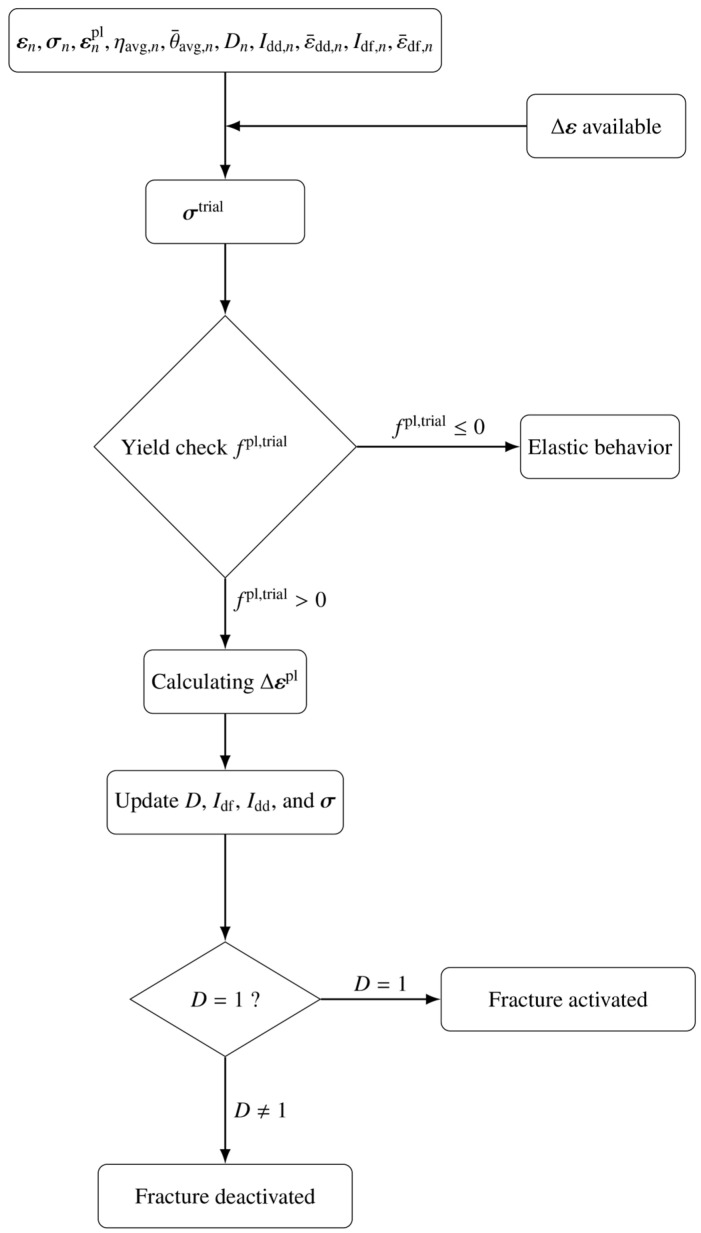
Flow chart of the implemented VUMAT user-subroutine in ABAQUS 2022.

The fracture criterion for the zirconium material was implemented as a VUMAT user-defined subroutine in ABAQUS 2022. A flow chart of the model implementation is given in Figure 7. To further validate the model, the plastic deformation and fracture behavior of various samples were simulated using an element deletion approach in ABAQUS/Explicit. The simulated force–displacement curves are shown in Figure 7 for different specimens. In each case, solid lines in three distinct colors represent experimental results from three parallel tests, while the corresponding simulated curve is depicted as a black dotted line. The simulation results indicate that the predicted force–displacement responses for CHR1 and SH samples exhibit strong agreement with experimental data, with predicted fracture points falling within the experimental range. For the NDBR6 sample, the predicted fracture initiation occurs slightly earlier than observed in all three experimental trials. In the case of the PSR2 sample, the predicted fracture displacement falls within the experimental range, although the predicted force is marginally higher than the experimentally recorded values. Although the MBW parameters were calibrated using stress-state data extracted at fracture initiation, it should be noted that the loading paths of several geometries, particularly CHR1 and SH, are distinctly non-proportional. The MBW formulation accounts for such effects through accumulated indicators, but the model assumes that deviations from proportionality can be captured by averaging the stress-state history. Consequently, fracture initiation predictions may be less accurate when the material undergoes rapid or discontinuous changes in stress triaxiality or Lode angle. This explains the slight discrepancy between the fitted DIL and the experimental data points for NDBR6 and PSR2. While the overall predictions are robust, these limitations demonstrate that non-proportional loading is a challenging aspect of stress-state-dependent fracture modeling.”

Overall, the simulated force–displacement curves demonstrate a high degree of consistency with experimental observations across all four specimen geometries, indicating that the fracture model has been effectively calibrated and is capable of accurately capturing the fracture behavior of zirconium under various loading conditions.

## 5. Fractography

The previous sections describe the investigations of the fracture behaviors of Zr at different stress states via experimental and numerical simulation methods. As a supplement for the fracture information, SEM was used to analyze the fracture surface, so the possible micro-damage mechanism can be explained.

**Figure 8:**
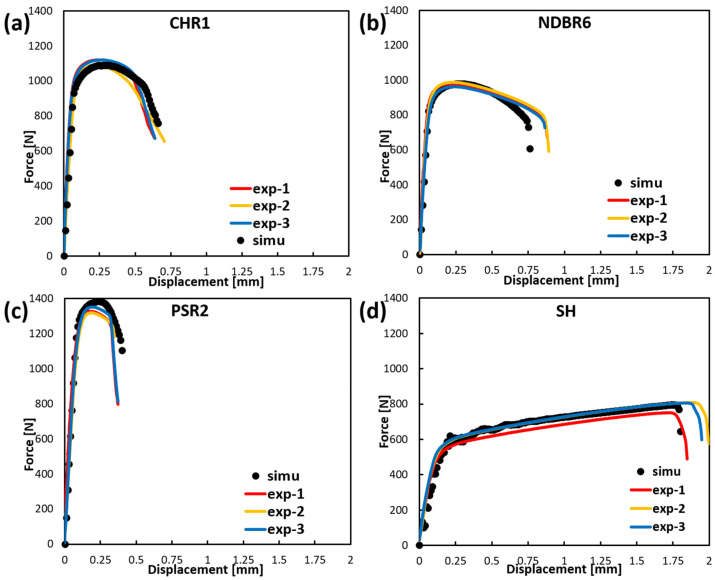
Finite element simulation results of the UT test on (**a**) CHR1, (**b**) NDBR6, (**c**) PSR2, and (**d**) SH samples.

**Figure 9:**
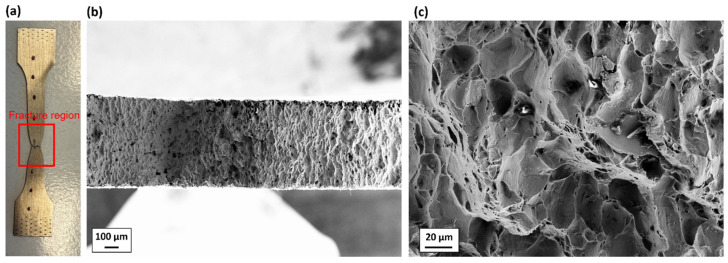
(**a**) One of the fractured SDB samples; (**b**,**c**) post-fracture morphology of it.

Figure 8 shows the post-fracture morphology of one of the tested SDB samples, which represents the uniaxial tensile state. Figure 9a shows the fracture sample and the region where the fractography is taken. Necking can be observed in this fracture sample, which is a typical feature of ductile fracture. Figure 9b,c show that the fracture surface of the SDB sample is full of dimples of different sizes, indicating that the micro-damage mechanism at the pure uniaxial tensile state, namely at the middle stress triaxiality range, the material failure is dominated by dimple fracture.

**Figure 10:**
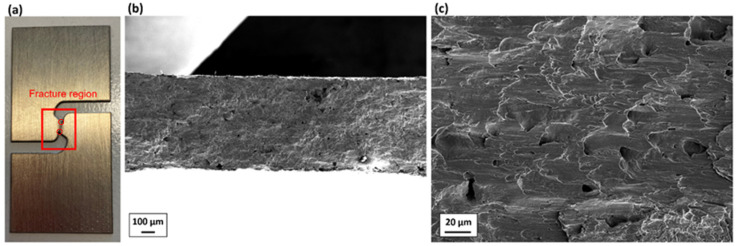
(**a**) One of the fracture SH samples; (**b**,**c**) post-fracture morphology of it.

Similarly, Figure 10 shows the post-fracture morphology of one of the tested SH samples, which represents the pure shear state. Figure 10a shows the fracture sample and the region where the fractography is taken. Shear can be observed at two tips of the crack (red circles in Figure 10a). Figure 10b,c show that numerous slip marks and minor dimples characterize the fracture surface. The fracture of the SH specimen, namely at a pure shear stress state, is dominated by shear stress.

Based on Figure 9 and Figure 10, it can be concluded that with increasing stress triaxiality, the micro-damage mechanisms of the investigated Zr shift from a shear-dominated mechanism to a tension-dominated mechanism.

In this study, the fracture behaviors of zirconium under relatively wide stress states are successfully investigated by testing samples with different geometries and reproduced by running numerical simulations. The fracture mechanisms of the pure shear and pure tension samples are analyzed. The primary goal of the study has been achieved. Still, there remain gaps for further improvements. Specifically, Charpy tests and compression tests should be carried out to cover negative and high stress triaxiality ranges. The fracture surfaces of more fractured samples, including Charpy and compression samples, should be analyzed to gain a deeper understanding of the failure mechanism as it transitions from negative to ultra-high stress triaxiality. These will be investigated in the future.

## 6. Conclusions

In this study, a hybrid experimental and numerical simulation approach is followed to investigate the fracture behavior of zirconium. In addition, the fracture surfaces of two fracture samples were analyzed using SEM. Several conclusions are summarized:

- Characterizing fracture behavior covering a wide range of stress states by conducting uniaxial tensile tests on samples with different geometries. Necking was observed in the SDB sample, which is a typical feature for ductile fracture. Shear was observed at two tips of the crack in the pure shear sample.
- The elasto-plasticity behavior of Zr was modeled using the Yoon2014 asymmetric yield function, and the MBW fracture model was successfully calibrated using stress-state-dependent data extracted from FE simulations. The calibrated model reproduced the experimental force–displacement response for all specimen types with good accuracy.
- The fractographic analysis of the fracture surface reveals that the micro-damage mechanisms of the investigated Zr shift from a shear-dominated mechanism to a tension-dominated mechanism with increasing stress triaxiality.
- From a practical standpoint, the combined macroscopic and microscopic findings highlight which stress-state regimes are most detrimental for zirconium components. Low stress triaxiality shear states favor shear-band localization and early fracture, while high-triaxiality plane-strain conditions promote void growth and rapid loss of load-carrying capacity. Avoiding geometric features or loading configurations that impose these critical stress states can therefore reduce the risk of premature fracture.

## Figures and Tables

**Table 1:** Basic mechanical properties and calibrated flow curve of zirconium.

Material	ρ	$\sigma_{yield}$	*E*	$\sigma_{UTS}$	*A*_80_	*ϑ* *
Zirconium	$6.52\;g/{cm}^{3}$	400 MPa	89 GPa	445 MPa	25%	0.34
Swift hardening law		$\sigma_{s}=A\cdot {\left({\bar{\varepsilon }}^{p}+B\right)}^{n}$				
Material	A	*B*	*n*			
Zirconium	646 MPa	0.0067	0.1			

* Refer to the work of Paredes and Wierzbicki [21].

**Table 2:** Local stress and strain values of the critical zirconium element when the fracture occurred.

Sample	$\eta_{avg}$	${\bar{\theta }}_{avg}$	PEEQ
CHR1	0.362	0.977	0.881
NDBR6	0.716	0.333	1.442
PSR2	0.589	0.106	0.428
SH	0.0362	0.0747	0.960

**Table 3:** Calibrated damage parameters for zirconium.

$D_{1}$	$D_{2}$	$D_{3}$	$D_{4}$
1	0.02098	0.9256	0.01

## Data Availability

The original contributions presented in this study are included in the article. Further inquiries can be directed to the corresponding author.

## Abbreviations

The following abbreviations are used in this manuscript:

MBW	Modified Bai-Wierzbicki model
SEM	Scanning electron microscope
LOCA	Loss of cooling agent
SDB	Standard dog bone
DIC	Digital image correlation
CHR1	Central hole samples with a radius of 1 mm
NDBR6	Notched-dog bone samples with a notch radius of 6 mm
PSR2	Plane strain samples with a radius of 2 mm
SH	Shear sample
SD	Strength differential
HCP	hexagonal close-packed
PEEQ	equivalent plastic strain
DIL	Ductile damage initiation locus
DFL	Ductile fracture locus
TolFun	Function convergence
TolX	Step size tolerance
